# Identifying At-Risk Patients with Combined Pre- and Postcapillary Pulmonary Hypertension Using Interventricular Septal Angle at Cardiac MRI

**DOI:** 10.1148/radiol.2018180120

**Published:** 2018-07-03

**Authors:** Christopher S. Johns, James M. Wild, Smitha Rajaram, Euan Tubman, David Capener, Charlie Elliot, Robin Condliffe, Athanasios Charalampopoulos, David G. Kiely, Andrew J. Swift

**Affiliations:** From the Academic Department of Radiology, Academic Unit of Radiology, Department of Infection, Immunity & Cardiovascular Disease, Magnetic Resonance Imaging Unit, University of Sheffield, Royal Hallamshire Hospital, Glossop Rd, Floor C, Sheffield S10 2JF, England (C.S.J., J.M.W., E.T., D.C., A.J.S.); Sheffield Pulmonary Vascular Disease Institute (C.E., R.C., A.C., D.G.K.) and Department of Radiology (S.R.), Sheffield Teaching Hospitals, Sheffield, England; and Insigneo Institute for In Silico Medicine, University of Sheffield, Sheffield, England (A.J.S.).

## Abstract

**Purpose:**

To assess interventricular septal (IVS) angle in the identification of combined pre- and postcapillary pulmonary hypertension (Cpc-PH) in patients with pulmonary hypertension (PH) due to left-sided heart disease.

**Materials and Methods:**

In this retrospective study, consecutive, incident patients suspected of having PH underwent same-day right-sided heart catheterization (RHC) and MRI at a PH referral center between April 2012 and April 2017. The diagnostic accuracy of the IVS angle to identify Cpc-PH in patients with pulmonary arterial wedge pressure (PAWP) greater than 15 mmHg was assessed by using receiver operator characteristic curves, sensitivity, specificity, and negative and positive predictive values. IVS angle also was assessed as a predictor of all-cause mortality by using Cox uni- and multivariable proportional hazards regression.

**Results:**

A total of 708 patients underwent same-day MRI and RHC, and 171 patients had PAWP greater than 15 mmHg. Mean age was 70 years (range, 21–90 years) (women: mean age, 69 years; range, 21–88 years) (men: mean age, 71 years; range, 43–90 years). Systolic IVS angle correlated with diastolic pulmonary gradient (DPG) (*r* = 0.739, *P* < .001). Receiver operating characteristic curve analysis showed septal angle enabled identification of Cpc-PH (DPG ≥ 7), with an area under the receiver operating characteristic curve of 0.911 (*P* < .001). A 160° threshold, derived from the first half of patients with raised PAWP, enabled identification of a DPG of at least 7 mmHg with 67% sensitivity and 93% specificity (*P* < .001) in the second cohort of patients with raised PAWP. IVS angle was predictive of all-cause mortality (standardized univariable hazard ratio, 1.615; *P* < .01).

**Conclusion:**

The systolic interventricular septal angle is elevated in patients with combined pre- and postcapillary pulmonary hypertension and enables one to predict those patients who have PH due to left-sided heart disease who have an increased risk of death.

Published under a CC BY 4.0 license.

*Online supplemental material is available for this article.*

See also the editorial by Rajiah in this issue.

## Introduction

Patients with left-sided heart disease commonly develop pulmonary hypertension (PH) (1), initially resulting from passive backward transmission of high left ventricular filling pressures through the pulmonary circulation. Some patients with postcapillary disease may subsequently develop a degree of precapillary vascular remodeling due to prolonged elevation of pulmonary arterial pressure (2–9). Previously, the difference between mean pulmonary arterial pressure (mPAP) and pulmonary arterial wedge pressure (PAWP), termed the transpulmonary gradient (TPG), was used to identify patients with PH out of proportion to left-sided heart disease. Subsequently, a study by Gerges et al (2) identified the diastolic pulmonary pressure gradient (DPG), which was calculated by subtracting mean PAWP from diastolic pulmonary artery pressure, as a superior prognostic parameter in patients with postcapillary disease. The Fifth World Symposium on Pulmonary Hypertension introduced the classification of combined pre- and postcapillary PH (Cpc-PH), defined as an mPAP level of at least 25 mmHg, a PAWP level greater than 15 mmHg, with either a DPG of 7 mmHg or more or pulmonary vascular resistance (PVR) greater than 3 WU (10). Patients with Cpc-PH are at greater risk for deterioration than are those with isolated postcapillary PH (Ipc-PH) but may benefit from PAH-specific therapy, especially in the context of randomized controlled trials (9,11,12) (*https://ClinicalTrials.gov* identifier, NCT02070991).

Previous noninvasive MRI techniques were used to measure left atrial volume index (13), echocardiographic parameters (14), or a combination of clinical, electrocardiographic, and echocardiographic features (6) and can be used to distinguish between pre- and postcapillary disease. However, there is currently no noninvasive method to identify patients likely to have Cpc-PH.

The aim of our study was to assess interventricular septal angle in the identification of Cpc-PH in patients with PH owing to left-sided heart disease.

We hypothesized that structural and functional imaging of the heart using cardiac MRI, specifically the interventricular septal angle, could enable differentiation of Cpc-PH from Ipc-PH.

## Materials and Methods

Ethical approval was granted by the local ethics committee, and institutional review board approval was attained for our retrospective study. Written informed consent was waived (ref c06/Q2308/8).

Consecutive incident patients suspected of having PH who underwent cardiopulmonary MRI at a PH referral center (15) from April 2012 to April 2017 were identified. All incident cases with MRI and right-sided heart catherization (RHC) on the same day were included. Our main study population comprised patients with left-sided heart disease diagnosed at RHC (PAWP > 15 mmHg). These patients were split into a derivation and validation cohort by date of imaging (October 1, 2016). The remaining incident patients with a normal PAWP were used as a comparison group to assess the underlying mechanism.

### Image Acquisition and Analysis

**Figure 1:**
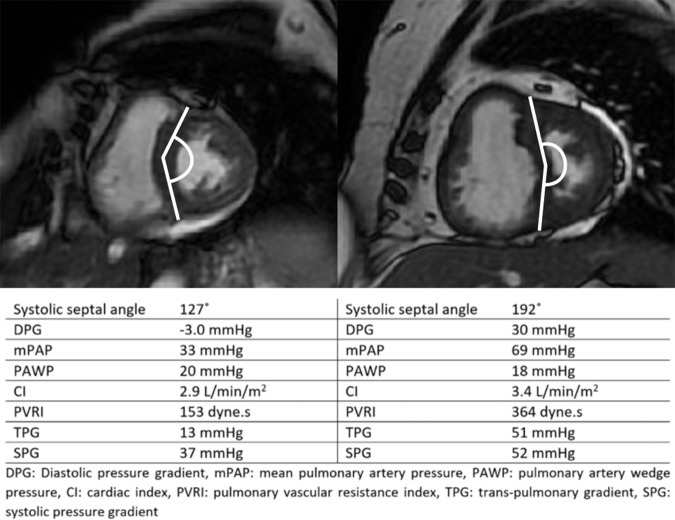
Representative images in a patient with a negative diastolic pulmonary pressure gradient and a normal septal angle (left) and a patient with an elevated diastolic pulmonary gradient (DPG) and a high septal angle (right).

All patients underwent cardiac 1.5-T MRI at the PH referral center as part of the routine clinical pathway. Short-axis steady state free-procession images were acquired with standard protocols, as previously described (16) and available in Appendix E1 (online). MR images were analyzed by a radiographer with 9 years of cardiac MRI experience (D.C.) on a GE Advantage Workstation 4.4 using GE Advantage Workstation ReportCard software (GE Healthcare, Milwaukee, Wis), with the observer blinded to all clinical information and results of other investigations. The interventricular septum (IVS) angle was measured as the angle formed between the insertion points of the ventricles to the midpoint of the septum, measured at end-systole (17–19). Figure 1 shows the IVS angle in one patient with high DPG and another with normal DPG. The standard cardiac contours and metrics were measured and calculated (16,20), as previously published. A description of these is available in Appendix E1 (online). These standard cardiac MRI metrics have previously been shown to have excellent interobserver reproducibility (21).

### RHC Procedure

RHC was performed at the PH referral center by using a balloon-tipped 7.5-French thermodilution catheter (Becton-Dickinson, Franklin Lakes, NJ) introduced via a Swann-Ganz catheter, usually via the internal jugular vein. Left-sided heart disease was defined as PAWP greater than 15 mmHg (10). DPG was calculated as diastolic pulmonary arterial pressure minus PAWP (4), with a DPG of 7 mmHg or higher considered diagnostic for combined pre- and postcapillary PH (10,22). Cardiac output was measured with thermodilution. PVR was defined as the difference of mPAP minus PAWP, which was then divided by cardiac output. Further analysis was performed by using PVR, with a threshold of greater than 3 Woods units (240 dyne·sec) to assess the IVS angle as a marker for Cpc-PH using both potential methods.

### Statistical Analysis

To test the accuracy of cardiac MRI parameters in the assessment of DPG, initial analysis was performed in all patients with PAWP greater than 15 mmHg. The Pearson correlation coefficient was calculated between each variable and DPG, transpulmonary gradient, and PVR. Scatterplots of each metric were interrogated to ensure linearity. To identify a suitable diagnostic threshold, the receiver operating characteristic curve was analyzed in the first half, and the Youden index was used to select a suitable threshold. In the second half of the patient cohort, the 2 × 2 contingency table and the χ^2^ test were used to determine sensitivity, specificity, and positive and negative predictive values for the threshold derived in the training cohort.

To assess the hemodynamic basis of the IVS angle as a marker of DPG, the Pearson correlation coefficient of septal angle and DPG with mean systolic and diastolic pulmonary arterial–to-systemic pressure ratios were analyzed. In the full cohort of cases, the IVS angle in each group of patients with PH (by cause [10]) was compared by using analysis of variance. The correlation coefficient between the septal angle against the DPG and the mean pulmonary arterial pressure was calculated for each group.

To assess the underlying mechanism for the IVS angle as a marker of the DPG, linear regression analysis of significant candidate MRI correlates was assessed. For linear regression analysis, the *z* score from the population with raised PAWP was calculated as follows: 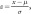, where µ is the mean and σ is the standard deviation, to enable comparison of the independent associations of MRI metrics with DPG. DPG was considered the dependent variable and any MRI metric with a significant correlation with DPG was considered a predictor.

Analysis of outcome was performed in the patients with increased PAWP. Our study period stretched from MRI to census (June 16, 2017) or all-cause mortality. Survival analysis was performed by using Cox proportional hazards regression analysis and the log-rank test at Kaplan-Meier curve analysis. For Cox regression, metrics were standardized with the *z* score. Kaplan-Meier plots were constructed by using recognized thresholds, when available. For IVS angle, the threshold from the Youden index in the first cohort of patients (160°) was used. Receiver operating characteristic curve analysis was used to predict death at 2 years from study enrollment, as this time point had a balance of patients who reached census (*n* = 122) and who died (*n* = 36). Survival analysis of six variables was performed; the Bonferroni correction was used, and *P* < .008 indicated a significant difference.

Reproducibility was assessed in 20 patients by a general radiologist (C.S.J.) with an interest in thoracic imaging and 6 years of experience, as per the standard reproducibility method, which is outlined in Appendix E1 (online).

Statistical analysis was performed by using SPSS, version 22 (IBM, Chicago, Ill) and GraphPad Prism 7 (GraphPad Software, San Diego, Calif). *P* <.05 indicated a significant difference, and data are presented as mean ± standard deviation, unless otherwise stated.

## Results

**Figure 2:**
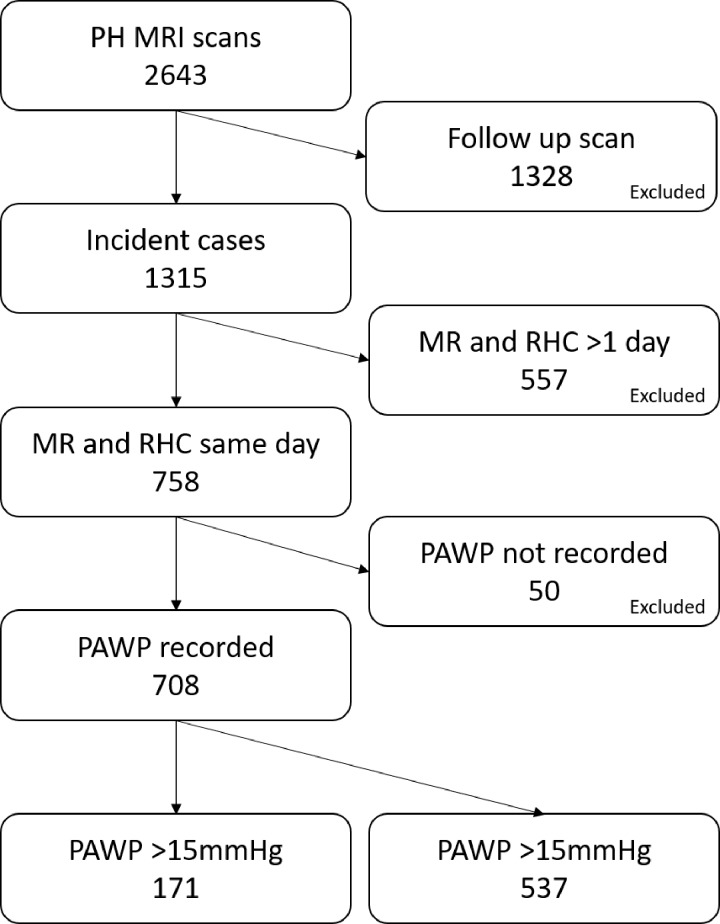
Flowchart shows patient inclusion criteria. *PAWP* = pulmonary artery wedge pressure, *PH* = pulmonary hypertension, *RHC* = right-sided heart catheterization.

In our study, 2643 patients underwent cardiopulmonary MRI. A total of 1315 patients underwent initial diagnostic imaging: of these, 758 underwent MRI and RHC on the same day. In 50 patients, PAWP was not recorded. A total of 171 patients had PAWP greater than 15. Figure 2 shows the algorithm for patient selection.

### Identification of High Diastolic Pulmonary Gradient

Mean age of patients was 70 years (range, 21–90 years), and 61% were women (mean age, 69 years; age range, 21–88 years) (men: mean age, 71 years; age range, 43–90 years). Fifty-six (33%) patients had a DPG of 7 mmHg or higher, 57 (33%) had a DPG of 0–6.9 mmHg, and 58 (34%) had a negative DPG. Table 1 presents the demographic and RHC data. Five patients did not have PH (all had DPG < 7 mmHg). A total of 103 patients had PH due to left-sided heart disease alone, 32 had combined pre- and postcapillary PH, 17 had coexistent lung disease, and 19 had coexistent chronic thromboembolic disease, as defined in the multidisciplinary team meeting. Left-sided heart disease was due to left ventricular diastolic dysfunction in 149 cases, left ventricular systolic dysfunction in nine, and valve disease in 13 (mitral regurgitation, *n* = 5; aortic stenosis, *n* = 5; aortic regurgitation, *n* = 1; mixed mitral and aortic disease, *n* = 1; unknown, *n* = 1). A total of 127 patients did not undergo vasodilator therapy, 31 received sildenafil alone, and 13 underwent dual therapy (sildenafil with macitentan, *n* = 8; bosentan, *n* = 4; ambrisentan, *n* = 1). In the Cpc-PH group, 13 patients had coexistent chronic thromboembolic PH, and nine had coexistent lung disease. Of these patients, 25 did not undergo vasodilator therapy, 19 received sildenafil alone, and 12 underwent dual therapy (sildenafil with macitentan, *n* = 8; bosentan, *n* = 4).

**Table 1:**
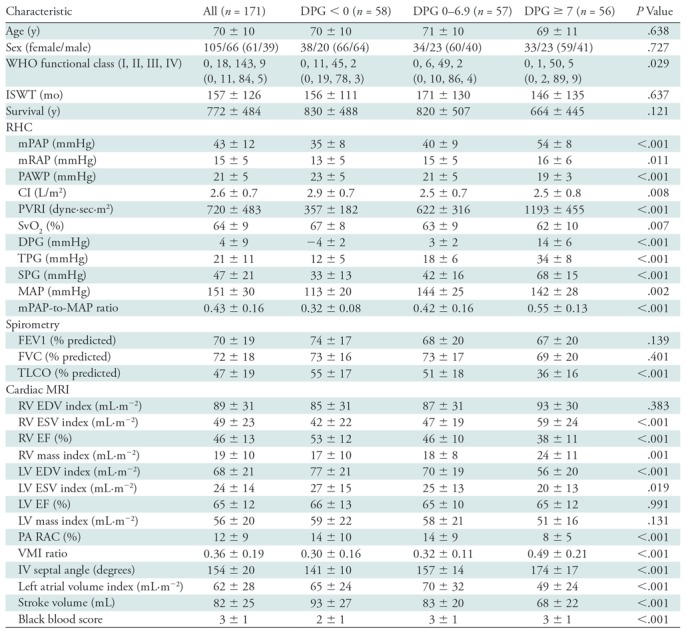
Baseline Patient Demographics, Hemodynamics, and Cardiac MRI Metrics in Patients with Pulmonary Arterial Wedge Pressure Greater than 15 mmHg, Split by the Diastolic Pulmonary Gradient

Note.—Continuous variables are presented as mean ± standard deviation. Categorical variables are presented as number, with percentage in parentheses. Analysis of variance was used to calculate significance of difference between groups for continuous variables, and χ^2^ test was for categorical variables. CI = cardiac index, DPG = diastolic pulmonary gradient, EDV = end-diastolic volume, EF = ejection fraction, ESV = end-systolic volume, FEV1 = forced expiratory volume in one second, FVC = forced vital capacity, ISWT = incremental shuttle walk test, IV = interventricular, LV = left ventricle, MAP = mean systemic arterial pressure, mPAP = mean pulmonary artery pressure, mRAP = mean right atrial pressure, PA = pulmonary artery, PAWP = pulmonary arterial wedge pressure, PVRI = pulmonary vascular resistance index, RAC = relative area change, RV= right ventricle, SPG = systolic pressure gradient (sPAP-PAWP), SvO_2_ = mixed venous oxygen saturations, TLCO = transfer factor for carbon monoxide, TPG = transpulmonary pressure gradient (mPAP-PAWP), VMI = ventricular mass index, WHO-FC = World Health Organization-Functional Class.

**Figure 3:**
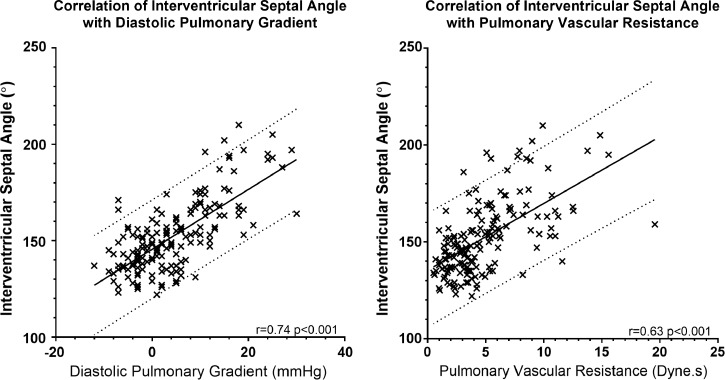
Scatterplots show the correlation of systolic interventricular septal angle with diastolic pulmonary pressure gradient and pulmonary vascular resistance. Solid line represents the line of best fit, and dotted lines represent 95% confidence intervals.

Systolic IVS angle correlated with DPG (*r* = 0.739, *P* < .001) and PVR (*r* = 0.626, *P* < .001). Figure 3 presents scatterplots of IVS angle against DPG and PVR. The correlation of IVS angle with DPG was greater in the group with elevated DPG (*R* = 0.190, *P* = .04 in patients with DPG < 7 mmHg; *R* = 0.549, *P* < .001 in patients with DPG ≥ 7 mmHg). Systolic IVS angle correlated with the transpulmonary gradient, PAWP, and mPAP (*r* = 0.77, *P* < .001; *r* = −0.20, *P* = .01; and *r* = 0.64, *P* < .001, respectively). Other MRI markers had weaker correlations with DPG; the correlations and *P* values for each metric with DPG, transpulmonary gradient, and PVR are provided in Appendix E1 (online). Patients with Cpc-PH had significantly elevated right ventricular end-systolic volumes and mass (and therefore ventricular mass index), reduced stroke volume, and right ventricular ejection fraction. In the Cpc-PH cohort, left ventricular end-diastolic volume and left atrial volume index were reduced, and the pulmonary arterial relative area change was significantly reduced.

Receiver operating characteristic curve analysis (Appendix E1 [online]) showed that septal angle could be used to identify patients with elevated DPG (≥7 mmHg) with an area under the receiver operating characteristic curve of 0.911 (*P* < .001). IVS angle also enabled identification of elevated pulmonary vascular resistance (>3 Woods units or >240 dyne·sec), with an area under the receiver operating characteristic curve of 0.783 (*P* < .001). In the first 85 patients (the derivation cohort), the Youden index was used to identify a septal angle of 160° as a diagnostic threshold for Cpc-PH, with 67% sensitivity and 93% specificity (*P* < .001). When tested in the second 86 patients (the validation cohort), the threshold of 160° had 67% sensitivity (95% confidence interval [CI]: 49, 81), 93% specificity (95% CI: 83, 97), 83% positive predictive value (95% CI: 64, 93), and 84% negative predictive value (95% CI: 64, 93).

In all patients suspected of having PH, there was a significant correlation between the IVS angle and the diastolic pulmonary arterial pressure or mPAP (Table 2). There was no correlation between the septal angle and either DPG or mPAP in patients without PH (*P* = .15 and *P* = .53, respectively). All of the groups had an elevated septal angle when compared with the groups of patients without PH. Patients with PH due to left-sided heart disease had a lower septal angle than did the other groups of patients with PH due to the combination of Ipc-PH and Cpc-PH.

**Table 2:**
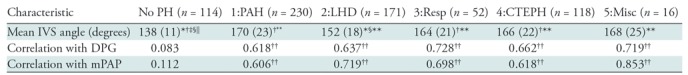
Assessment of Systolic Interventricular Septal Angle in Patients with or without Pulmonary Hypertension

Note.—Significant differences are defined as *P* < .05 after Bonferroni correction. CTEPH = chronic thromboembolic pulmonary hypertension, DPG = diastolic pulmonary gradient, LHD = left-sided heart disease, Misc = pulmonary hypertension due to miscellaneous causes, mPAP = mean pulmonary arterial pressure, PAH = pulmonary arterial hypertension, PH = pulmonary hypertension, Resp = respiratory.

*Different to group 1:PAH.

^†^ Different to group 2:LHD.

^‡^ Different to group 3:lung disease.

^§^ Different to group 4:CTEPH.

^||^ Different to group 5:misc.

**Different to no PH.

^††^ Correlation is statistically 21significant.

### Hemodynamic Basis of Interventricular Septal Angle

Table 3 shows the correlation between IVS angle and DPG with the ratios of mean, diastolic, and systolic pulmonary artery to systemic pressure. Although IVS angle and DPG correlated with all three, the strongest correlation was for systolic pulmonary arterial pressure–to–systolic systemic pressure ratio (*R* = 0.674 for IVS angle, *R* = 0.702 for DPG).

**Table 3:**
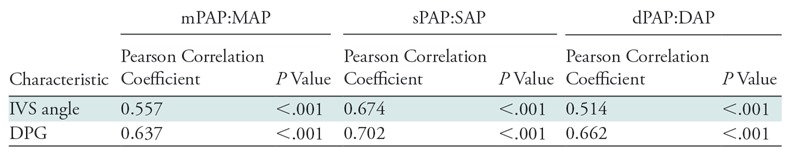
Pearson Correlation Coefficients between Hemodynamic Measures in the Whole Cohort

Note.—DAP = diastolic systemic blood pressure, dPAP = diastolic pulmonary arterial pressure, DPG = diastolic pulmonary gradient, IVS = interventricular septum, MAP = mean systemic arterial pressure, mPAP = mean pulmonary arterial pressure, PAWP = pulmonary arterial wedge pressure, sPAP = systolic pulmonary arterial pressure, SAP = systemic systolic blood pressure.

Systolic IVS angle had modest correlations with right ventricular end-diastolic volume (*R* = 0.257, *P* = .001), right ventricular end-systolic volume (*R* = 0.302, *P* < .001), left ventricular end-diastolic volume (*R* = −0.231, *P* = .002), left ventricular end-systolic volume (*R* = −0.140, *P* = .07), and the ratio of right-to-left end-diastolic volume (*R* = 0.420, *P* < .001) and end-systolic volume (*R* = 0.462, *P* < .001). At multivariable analysis, IVS angle was independently associated with DPG (hazard ratio, 11.807; 95% CI: 9.7, 13.9) and right-to-left end-systolic volume ratio (hazard ratio, 3.400; 95% CI: 1.3, 5.5).

### Prediction of Outcome

**Figure 4:**
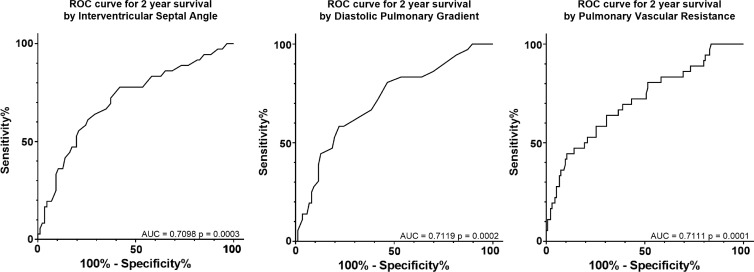
Receiver operating characteristic *(ROC)* curves used to predict death at 2 years by using interventricular septal angle, diastolic pulmonary gradient, and pulmonary vascular resistance. *AUC =* area under the ROC curve.

Mean follow-up was 2.1 years (standard deviation, 1.3), during which there were 48 deaths. Systolic IVS angle enabled prediction of all-cause mortality at univariable analysis, with a standardized Cox proportional hazard ratio of 1.615 (95% CI: 1.253, 2.082; *P* < .001). Table 4 shows the univariate hazard ratios for outcome. Kaplan-Meier analysis, dichotomized by 160°, showed a difference in outcome (log-rank test, χ^2^ = 11.02; *P* < .001) and is available in Appendix E1 (online). DPG, PVR, and transpulmonary gradient were indicative of death (standardized Cox proportional hazard ratio, 1.708, 1.667, and 1.609 respectively; *P* < .008). IVS angle, DPG, and PVR were all used to predict death at 2 years (area under the curve = 0.71 for all three factors, *P* < .001) (Fig 4).

**Table 4:**
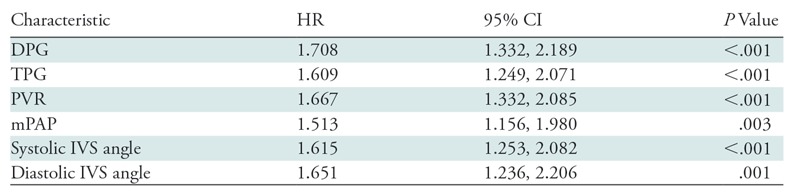
Univariable Cox Proportional Hazards and 95% CI for Diastolic Pulmonary Gradient, Transpulmonary Gradient, Pulmonary Vascular Resistance, and Systolic Interventricular Septum Angle Standardized by *z* Score

Note.—CI = confidence interval, DPG = diastolic pulmonary gradient, HR = hazard ratio, IVS = interventricular septum, mPAP = mean pulmonary arterial pressure, PVR = pulmonary vascular resistance, TPG = transpulmonary gradient.

At univariable analysis, PAH vasodilator therapy was associated with death (Cox univariable hazard ratio, 1.83; 95% CI: 1.026, 3.266; *P* = .04) (log-rank χ^2^ = 4.314, *P* = .04). However, when entered as a dummy variable with septal angle or hemodynamic parameters, it did not reach statistical significance (*P* = .26).

### Reproducibility of the Interventricular Septal Angle Measurement

There was excellent interobserver agreement (intraclass correlation coefficient= 0.902, *P* < .001). A Bland-Altman plot showed a tiny bias of −2% and close 95% limits of agreement of −12% to 8%. There was also excellent intraobserver agreement (intraclass correlation coefficient = 0.979, *P* < .001). Bland-Altman analysis showed a tiny bias of 2% and 95% limits of agreement of −4% to 8%. Bland-Altman plots for intra- and interobserver reproducibility are provided in Appendix E1 (online).

## Discussion

Our study shows that IVS angle can be used to identify Cpc-PH in patients with PAWP greater than 15 mmHg who are referred to a PH center. A systolic IVS angle of 160° enabled identification of patients with elevated DPG with 67% sensitivity and 93% specificity and served to identify patients with a higher risk of death.

We postulate that IVS angle represents a good estimate for the transpulmonary gradient, as it is a marker of the pressure difference between the left and right ventricle (23). However, it should be noted that this is different from the DPG, which is the pressure differential between the pulmonary artery and the mean PAWP (a surrogate marker for left atrial pressure). We have shown that the ratio of pressures between the pulmonary artery and systemic circulations are accurately reflected by the IVS angle, particularly at systole. We have also shown a strong relationship between the pulmonary arterial–to–systemic pressure ratio and DPG. The association between IVS flattening and DPG is independent of other cardiac volumetric and functional measurements. However, the relative volumes of the right and left ventricle do contribute to a lesser extent. While there is modest correlation between IVS angle and right-to-left ventricular volume ratio, the strongest correlation is with DPG, suggesting the flattening is most associated with the pressure gradient between the right and left ventricles.

Patients with Cpc-PH had features of precapillary PH at cardiac MRI, such as significantly elevated right ventricular end-systolic volume and mass (and thus ventricular mass index), reduced stroke volume, and right ventricular ejection fraction. In the Cpc-PH cohort, left ventricular end-diastolic volume and left atrial volume index were reduced (reduced filling). In support of the model that precapillary pulmonary vascular remodeling caused reduced precapillary arterial compliance in the patients with Cpc-PH, pulmonary arterial relative area change was significantly reduced. These findings suggest the presence of precapillary PH in patients with left-sided heart disease; however, the strongest metric was systolic IVS angle. Systolic IVS angle is easily measured and has excellent inter- and intraobserver reproducibility. Systolic IVS angle was the most diagnostic of Cpc-PH, likely because this is when the pressure difference between the right and left ventricle is most marked.

There is ongoing debate regarding the best way to assess for Cpc-PH. Initially, the transpulmonary gradient (mPAP-PAWP) was used to identify patients who had a precapillary component to their PH (4). Because this is affected by cardiac output, PVR, and PAWP, it is now recommended that a DPG of 7 mmHg or higher, a PVR of more than 3 Woods units, or both should be used to define Cpc-PH (22,25). The IVS angle correlated more strongly with DPG than with PVR, likely because it is a surrogate measure for pressure differences rather than for the vascular resistance, which is also related to cardiac output. There is debate over the importance of negative DPG, with reports of negative DPG being associated with a favorable outcome rather than a representation of the inaccuracies of PAWP measurement (26). Negative DPG is likely due to use of mean PAWP in DPG calculation rather than to diastolic PAWP. Further work is warranted to identify the most robust hemodynamic marker for Cpc-PH.

Current recommendations for PH due to left-sided heart disease center on treatment of the underlying left-sided heart disease; for example, correcting valvular heart disease, optimizing volume status, or heart failure therapy. However, there is interest in potential pulmonary vasodilator therapy. A noninvasive tool, such as IVS angle, to differentiate Cpc-PH from Ipc-PH for inclusion in potential clinical trials would be beneficial (9–12,24,27,28). Furthermore, in patients suspected of having PH due to left-sided heart disease, a flattened IVS angle should trigger a search for potential causes of precapillary disease.

Our study was limited by its retrospective design and the fact that it was conducted in a PH referral center, where the incidence of PH is higher than in the general population of patients with left-sided heart disease. Furthermore, the proposed threshold of IVS angle to identify patients with high diastolic pressure gradients requires assessment in a separate cohort. Future work is required to evaluate this threshold, assess the role of septal measurement in a more general cohort of patients with left-sided heart disease, and assess similarities with idiopathic pulmonary arterial hypertension.

In conclusion, MRI-derived systolic IVS angle can be used to noninvasively identify patients with combined pre- and postcapillary pulmonary hypertension in a cohort of patients suspected of having PH, and elevated PAWP. IVS angle can be used to predict which patients with left-sided heart disease are at risk for a poor outcome and may enable us to identify patients for targeted therapy.

**Summary**

The systolic interventricular septal angle measured at MRI is elevated in patients with combined pre- and postcapillary pulmonary hypertension in patients with left-sided heart disease; the interventricular septal angle enables one to predict which patients are at risk for a poor outcome.

**Implications for Patient Care**

■ Patients with combined pre- and postcapillary pulmonary hypertension are at risk for a poor outcome and may benefit from targeted pulmonary vascular therapy.
■ The interventricular septal angle measured with MRI is elevated in patients with combined pre- and postcapillary pulmonary hypertension.
■ The interventricular septal angle measured with MRI enabled prediction of patients who have an increased risk of death.

## APPENDIX

Appendix E1, Table E1 (PDF)

## SUPPLEMENTAL FIGURES

Figure E1:

Figure E2:

Figure E3:
