# Supplementary material for: Identifying At-Risk Patients with Combined Pre- and Postcapillary Pulmonary Hypertension Using Interventricular Septal Angle at Cardiac MRI
Source: Radiology. 2018 Jul 3;289(1):61–8. doi: 10.1148/radiol.2018180120 (PMC6190488; doi:10.1148/radiol.2018180120)
Supplement: Appendix E1, Table E1 (PDF) [file ry180120suppa1.pdf]

©RSNA, 2018
10.1148/radiol.2018180120

Appendix E1

Cardiac MRI Parameters

MRI was performed on a GE HDx 1.5-T whole body scanner (GE Healthcare, Milwaukee, Wisconsin), with the patient supine, using an 8-channel cardiac coil. Short axis (SA) cine images were acquired using a retrospectively cardiac gated multisection steady-state free precession (SSFP) sequence during a breath-hold, with 20 phases per cardiac cycle. A stack of axial images with section thickness of 8 mm with a 2 mm intersection gap or 10 mm with no intersection gap was acquired, covering both ventricles from base to apex. The SSFP sequence parameters were: TR 2.8 ms, TE 1.0 ms, flip angle 50°, field of view 48 × 43.2, 256 × 256 matrix and 125 kHz bandwidth. Phase contrast imaging was also performed in the pulmonary artery and the ascending aorta with the following parameters: TR 5.6 ms, TE 2.7 ms, section thickness 10 mm, FOV 48 × 28.8, bandwidth 62.5 kHz, matrix 256 × 128 and velocity encoding 150 cm/s. The Q flow images were retrospectively ECG gated with 40 phases. Axial black blood imaging was performed using a dual inversion recovery fast spin echo sequence, with a stack of 8 mm sections with 10 mm spacing taken through the long axis plane of the pulmonary artery.

Image Analysis

Routinely measured cardiac MI metrics included the left and right ventricular end-diastolic volume, end-systolic volumes, right and left ventricular stroke volume and mass (all indexed to body surface area), right and left ventricular ejection fractions and ventricular mass index (RV mass divided by LV mass). Maximal and minimal PA areas were manually traced, and relative area change was defined by the following equation: RAC = (maximum area-minimum area)/minimum area (22). Pulmonary artery average velocity was calculated as QFlow forward PA velocity/diastolic PA size. Black blood slow flow artifact was scored using a semiquantitative scale from 0 to 5 (0 = absent, 1 = segmental, 2 = lobar, 3 = distal main, 4 = proximal main and 5 = trunk) (23).

Reproducibility Methodology

To assess the reproducibility of the IVS angle measurement, a second reviewer (CSJ, a general radiologist with an interest in thoracic imaging with 6 years experience) analyzed the IVS angle in the first consecutive 20 patients with a raised pulmonary arterial wedge pressure, blinded to the previous analysis and any clinical details. In a separate sitting seven days later, the same 20 patients were reanalyzed by CSJ. The intra and interobserver reproducibility were assessed with intra class coefficient (using two-way mixed absolute agreement) and construction of Bland-Altman graphs.

Figure E1: ROC analysis for Septal angle to assess for an elevated DPG or PVR as the diagnosis of Cpc-PH.

Figure E2: Kaplan Meier analysis of patients with elevated PAWP dichotomized by interventricular septal angle, diastolic pulmonary gradient and pulmonary vascular

resistance. The interventricular septal angle does not have a published threshold so the diagnostic threshold identified in this paper is used.

Figure E3: Bland-Altman plots of inter and intraobserver agreement for systolic interventricular septal angle.

Table E1: Cardiac MRI Metrics Linear Correlations with DPG, TPG and PVR

	DPG		TPG		PVR	
	r	*P* value	r	*P* value	r	*P* value
Systolic IVS angle	0.739	<0.001	0.772	<0.001	0.626	<0.001
Diastolic IVS angle	0.534	<0.001	0.534	<0.001	0.575	<0.001
RVEDVI	0.118	0.124	0.095	0.219	0.079	0.304
RVESVI	0.356	<0.001	0.219	0.004	0.279	<0.001
RVEF	–0.550	<0.001	–0.516	<0.001	–0.469	<0.001
RVSVI	–0.297	<0.001	–0.28	<0.001	–0.262	0.001
LVEDVI	–0.398	<0.001	–0.296	<0.001	–0.287	<0.001
LVESVI	–0.205	0.007	–0.194	<0.001	–0.162	0.034
LVEF	–0.033	0.668	0.046	0.546	0.000	1.000
LVSVI	–0.441	<0.001	–0.283	<0.001	–0.304	<0.001
RV mass index	0.323	<0.001	0.333	<0.001	0.293	<0.001
LV mass index	–0.249	0.001	0.127	0.098	–0.247	0.001
LA volume index	–0.326	<0.001	–0.257	0.001	–0.206	0.007
Systolic PA area	0.301	<0.001	0.348	<0.001	0.254	0.001
Diastolic PA area	0.364	<0.001	0.422	<0.001	0.329	<0.001
PA RAC	–0.230	0.003	–0.288	<0.001	–0.285	<0.001
Black Blood Score	0.432	<0.001	0.405	<0.001	0.455	<0.001
VMI	0.500	<0.001	0.465	<0.001	0.463	<0.001

RVEDVI = right ventricular end-diastolic volume index, RVESVI = right ventricular end-systolic volume index, RVEF = right ventricular ejection fraction, RV = right ventricle, LVEDVI = left ventricular end-diastolic volume index, LVESVI = left ventricular end-systolic volume index, LVEF = left ventricular ejection fraction, PA RAC = pulmonary artery relative area change, VMI = ventricular mass index, IVS = interventricular septum, DPG = diastolic pulmonary gradient, TPG = trans pulmonary gradient, PVR = pulmonary vascular resistance.
