# Supplementary figures and images for: Identifying At-Risk Patients with Combined Pre- and Postcapillary Pulmonary Hypertension Using Interventricular Septal Angle at Cardiac MRI
Source: Radiology. 2018 Jul 3;289(1):61–8. doi: 10.1148/radiol.2018180120 (PMC6190488; doi:10.1148/radiol.2018180120)

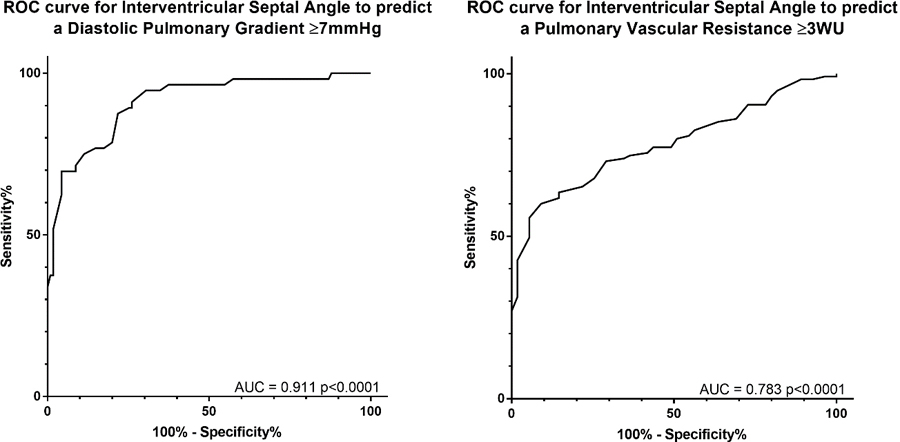

Supplement: Figure E1: [file ry180120suppf1.jpg]

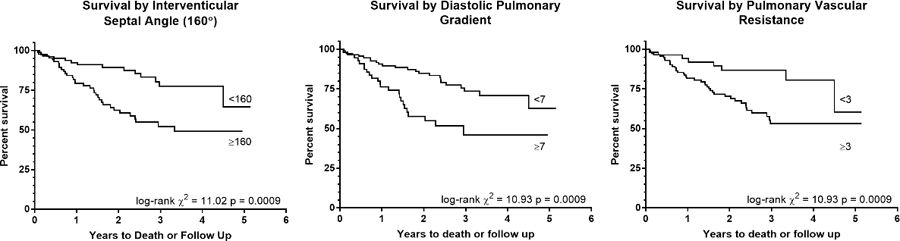

Supplement: Figure E2: [file ry180120suppf2.jpg]

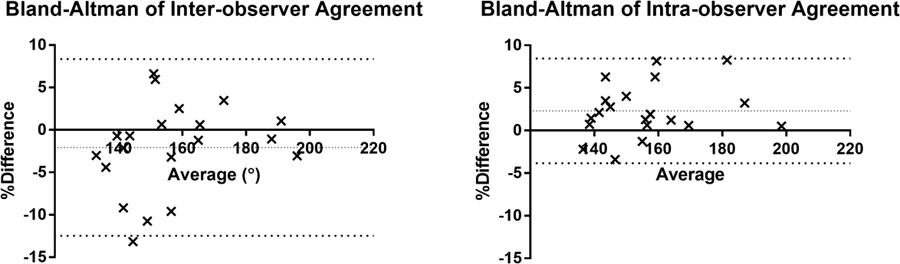

Supplement: Figure E3: [file ry180120suppf3.jpg]
